# Temporary ectropion therapy by adhesive taping: a case study

**DOI:** 10.1186/1746-160X-4-12

**Published:** 2008-07-21

**Authors:** Thomas Schrom, Anke Habermann

**Affiliations:** 1 Department of Oto-Rhino-Laryngology, Helios Clinics Bad Saarow, Germany; 2 Department of Ophthalmology, Martin-Luther-University Halle-Wittenberg, Germany

## Abstract

**Introduction:**

Various surgical procedures are available to correct paralytic ectropion, which are applied in irreversible facial paresis. Problems occur when facial paresis has an unclear prognosis, since surgery of the lower eyelid is usually irreversible. We propose a simple method to correct temporary ectropion in facial palsy by applying an adhesive strip.

**Patients and methods:**

Ten patients with peripheral facial paresis and paralytic ectropion were treated with an adhesive strip to correct paralytic ectropion. We used "Steri-Strips" (45 × 6.0 mm), which were taped on the carefully cleaned skin of the lower eyelid and of the adjacent zygomatic region until the prognosis of the paresis was clarified. In addition to the examiner's evaluation of the lower lacrimal point in the lacrimal lake, subjective improvement of the symptoms was assessed using a visual analogue scale (VAS, 1–10).

**Results:**

9 patients reported a clear improvement of the symptoms after adhesive taping. There was a clear regression of tearing (VAS (median) = 8; 1 = no improvement, 10 = very good improvement), the cosmetic impairment of the adhesive tape was low (VAS (median) = 2.5; 1 = no impairment, 10 = severe impairment) and most of the patients found the use of the adhesive strip helpful. There was slight reddening of the skin in one case and well tolerated by the facial skin in the other cases.

**Conclusion:**

The cause and location of facial nerve damage are decisive for the type of surgical therapy. In potentially reversible facial paresis, procedures should be used that are easily performed and above all reversible without complications. Until a reliable prognosis of the paresis can be made, adhesive taping is suited for the temporary treatment of paralytic ectropion. Adhesive taping is simple and can be performed by the patient.

## Introduction

Functional symptoms in peripheral facial paresis are especially due to the malfunction of both facial sphincter systems, the orbicularis oris and the orbicularis oculi muscle. A paretic orbicularis oculi muscle causes clinically visible lagophthalmos that can lead to varying degrees of keratopathy and thus to the loss of vision. Dropping of the eyebrow, secondary dermato- or blepharochalasis of the upper lid and paralytic ectropion can also result in addition to lagophthalmos [1,2]. In paralytic ectropion, the nasolacrimal system is considerably impaired by the migration of the lacrimal point out of the lacrimal lake. Closure of the eyelid is normally a complex process. It begins with the lowering of the upper eyelid in the vertical direction and makes short rapid horizontal movements in the medial direction. The lower lid, on the other hand, is pushed up and moved more strongly in the medial direction, in which the lid opening is shortened by 1 to 2 mm. The medial movement of the lids promotes the locomotion of the lacrimal fluid to the lacrimal lake, the lacrimal point and lacrimal canal and acts as a suction and force pump. The malfunction of the orbicularis oculi muscle in peripheral facial paresis can lead to both lagophthalmos with the risk of corneal desiccation and to a sensory disorder of the nasolacrimal system caused by the loss of the lacrimal pump.

The cause, location and prognosis of facial nerve damage are decisive for the type of surgical rehabilitation [3], although the surgical indication should be carefully made in the case of reversible paresis. Moreover, methods that are simple and reversible without complications should be used [4]. While different reversible surgical methods (including implants) exist for correcting lagophthalmos [5], the situation in the lower lid is considerably more difficult. A number of different surgical procedures are available for eliminating paralytic ectropion [2,6-9]. Common methods include blepharorrhaphy (either medial or lateral depending on the finding), canthoplasty, lateral bridle grafts, and different types of tarsus excision [7]. The above-mentioned methods result in a horizontal lifting of the lower lid by an irreversible shortening of the eyelid. Furthermore, bridle grafts from the temporal muscle or alloplastic material [10] and augmentation of the lower lid tarsus with cartilage or alloplastic materials (e.g. porous polyethylene) are also used to lift the lower lid edge [2,11]. Except for the transfer of the temporal muscle, the other procedures are of a purely static nature and ultimately serve to bring the lower lid closer to the bulb and to relocate the lower lacrimal point in the lacrimal lake. Surgical correction of the lost suction and force pump of the lid has thus far not been possible.

There have hardly been any descriptions in the literature of conservative treatment procedures for correcting paralytic ectropion. One possible method is temporary correction by taping the lower lid to the adjacent zygomatic region with adhesive strips to invert the lower lacrimal point into the lacrimal lake. Adhesive strips have only been used in individual cases to correct lagophthalmos, entropion or ptosis of the eyebrow [12-15]. There have thus far been no systematic examinations of the correction of paralytic ectropion in a patient population for acceptance of this procedure as a conservative treatment method.

## Patients and methods

**Figure 1:**
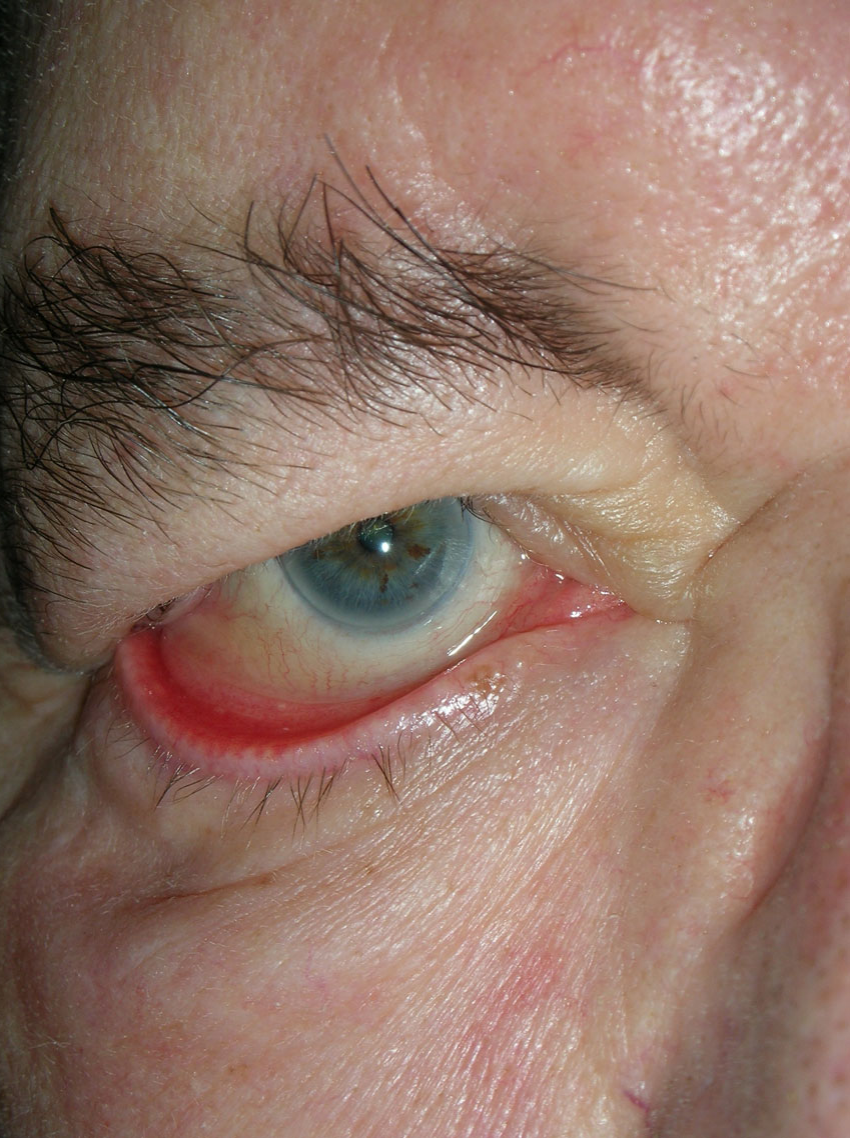
Ectropion in facial palsy.

**Figure 2:**
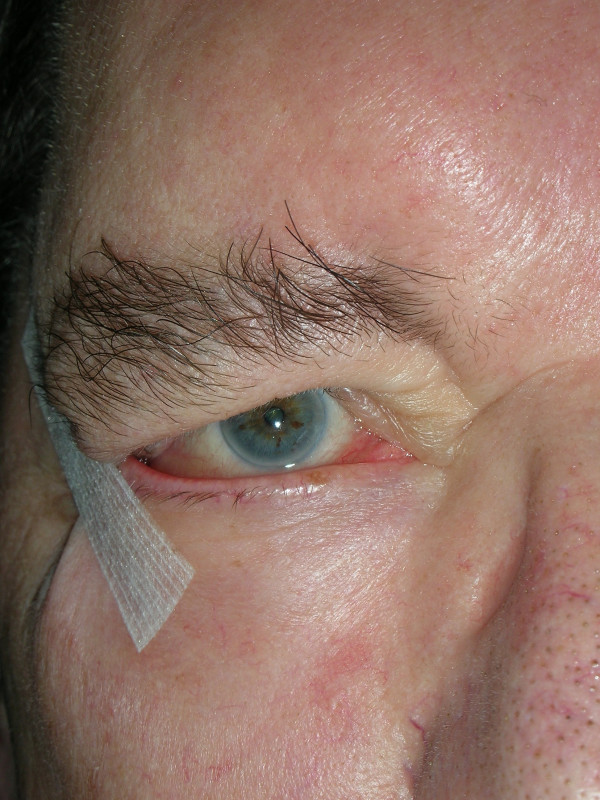
Ectropion corrected by applying adhesive tape.

In a total of 10 patients suffering from peripheral facial paresis with resultant lagophthalmos and ectropion, taping of the lower lid to the adjacent zygomatic region with adhesive strips was performed either until the nerve completely recovered or final surgery of the ectropion. The patient population consisted of 5 females and 5 males with a mean age of 69.3 years. Facial paralysis resulted after resection of an acoustic neurinoma in 4 cases and after resection of a parotid tumor, temporal bone fracture, cholesteatoma and 3 cases of zoster oticus. The mean follow-up time was 3 months. Fig. 1 shows paralytic lower lid ectropion and fig. 2 the findings after adhesive strip taping. Steri-strips (45 × 6.0 mm) were attached between the lower lid and adjacent zygomatic region.

The pretherapeutic examination of ectropion includes an evaluation of the position of the lower lid in relation to the eyeball, horizontal palpebral fissure and lower lid tension. Lower lid tension can be assessed with the so-called snap test and distraction test. In the snap test the lower lid is pulled down then released so it can rebound. A lid that has not previously been operated on should return to its original anatomical position within one or two seconds. A delayed reaction is a sign of a loss of elasticity. In the distraction test, the lower lid is held between two fingers and gently pulled in the ventral direction. Lifting of the lower lid from the eyeball by more than 8 mm is considered pathological and is pathognomonic for atonia of the lower lid. In addition to the evaluation of the lower lacrimal point in the lacrimal lake, the patient makes a subjective assessment using a visual analog scale (VAS 1–10). The patient can rate the reduction in tearing (1 = no improvement, 10 = excellent improvement), cosmetic impairment (1 = no impairment, 10 = considerable impairment) and practicability (1 = not helpful, 10 = very helpful). In all patients, a lid implant (platinum chain) was pretarsally implanted to correct the paralytic lagophthalmos.

## Results

**Figure 3:**
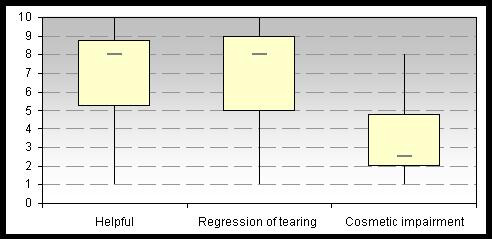
Results of the visual analog scales.

After fixation of the adhesive strips, inversion of the lower lacrimal point into the lacrimal lake was observed in all patients. Nine patients reported a clear improvement in their symptoms after application of the adhesive tape. There was a clear reduction in tearing (VAS (median) = 8; 1 = no improvement, 10 = excellent improvement), little cosmetic impairment (VAS (median) = 2.5; 1 = no impairment, 10 = considerable impairment) and most patient found the adhesive bridle to be helpful (VAS (median) = 8; 1 = not helpful, 10 = very helpful). In the meantime, 5 patients use the adhesive tape daily and 4 patients occasionally depending on the situation. Fig. 3 illustrates the results of the visual analog scales.

In one case, there was slight reddening of the skin, which completely healed after the adhesive bridle was no longer used. No other complications were observed.

## Discussion

The causes of ectropion may be age-related, paralytic, cicatricial, mechanical or hereditary. It may also be caused by tumor infiltration in the infra-orbital region. Depending on the cause, different surgical procedures are used to correct the ectropion [16], which include in the most cases a shortening of the palpebral fissure for horizontal lifting of the lower lid or the augmentation of the lower lid tarsus with cartilage or alloplastic materials (e.g. porous polyethylene) [7,11]. Since the reversibility of these procedures can be problematic, the indication to use the above methods should be carefully made in potentially reversible paresis [4]. The clinical course should be confirmed with electrophysiological tests in doubtful cases and surgical intervention initially postponed in the case of unclear findings.

In addition to lagophthalmos, manifestations of facial paresis around the eye include a dropping eyebrow, secondary dermato- or blepharochalasis of the upper lid and ectropion [1,2], in which the paralytic ectropion mainly affects the lateral parts of the lower lid in most cases [10,17]. Taping with adhesive strips has been described as a conservative therapeutic approach for both lagophthalmos and ptosis of the eyebrow [12-14]. Our study has shown that taping the lower lid to the adjacent zygomatic region with adhesive strips leads to a static lifting of the lower lid and to inversion of the lower lacrimal point into the lacrimal lake. This considerably reduced the patients' subjective symptoms. The method is thus suitable for both temporary conservative therapy and for patients who decline further surgery. The use of skin-colored Steri-strips (45 × 6.0 mm) reduced cosmetic impairment even further. The advantages of the Steri-strips are good skin tolerance, wide availability in most hospitals and the low cost. Their application is easy and can be performed by the patient, if necessary.

## Conclusion

The patients' subjective well-being could be improved overall using adhesive strips. The correction of paralytic ectropion by adhesive strips is especially suited as a temporary, conservative procedure. The method is simple, inexpensive and can be performed by the patient.

## Competing interests

The authors declare that they have no competing interests.

## Authors' contributions

TS conceived the study and drafted the manuscript. AH participated in the design of the study, acquisition of the data and statistical analysis. All authors read and approved the final manuscript.
